# The Influence of Osteoporosis and Diabetes on Dental Implant Stability: A Pilot Study

**DOI:** 10.3390/medicina61010074

**Published:** 2025-01-04

**Authors:** Liliana Sachelarie, Ioana Scrobota, Felicia Cioara, Timea Claudia Ghitea, Corina Laura Stefanescu, Liana Todor, Georgiana Ioana Potra Cicalau

**Affiliations:** 1 Department of Preclinical Discipline, Faculty of Dental Medicine, Apollonia University, 700511 Iasi, Romania; 2 Department of Dental Medicine, Faculty of Medicine and Pharmacy, University of Oradea, 10 1st Decembrie Street, 410073 Oradea, Romania; fcioara@uoradea.ro (F.C.); timea.ghitea@csud.uoradea.ro (T.C.G.); liana.todor@uoradea.ro (L.T.); cicalau.georgiana@uoradea.ro (G.I.P.C.); 3 Faculty of Medicine and Pharmacy, University Ovidius, Constanta, Mamaia Boulevard, 900527 Constanta, Romania; corina.stefanescu@yahoo.com

**Keywords:** dental implants, osteoporosis, diabetes, implant stability, osseointegration, Periotest, Osstell ISQ

## Abstract

*Background and Objectives*: Dental implants are a reliable solution for tooth loss; however, systemic conditions like osteoporosis and diabetes, which affect bone quality, healing, and stability, underline the importance of understanding their impact on enhanced outcomes. This study evaluated the comparative effects of osteoporosis and diabetes on dental implant stability over 12 months, utilizing objective implant mobility and stability measures. *Materials and Methods*: This prospective cohort study involved 50 patients, divided into 21 with type 2 diabetes and 29 with osteoporosis, with implant stability assessed at 6 and 12 months using Osstell ISQ and Periotest M devices and statistical analysis identifying differences between groups and time intervals at a significance level of *p* < 0.05. *Results*: At 6 months, the osteoporosis group showed higher initial stability (mean ISQ: 67.04 ± 5.42) compared to the diabetes group (mean ISQ: 62.10 ± 5.46; *p* = 0.0215)), and by 12 months, both groups showed significant improvements in ISQ scores (osteoporosis: 68.93 ± 4.83; diabetes: 65.79 ± 3.87), with Periotest values indicating more significant reductions in implant mobility, particularly in diabetic patients (osteoporosis: −3.34 ± 1.59; diabetes: −2.81 ± 1.44; *p* = 0.0001). *Conclusions*: Osteoporosis and diabetes significantly impact implant stability through distinct osseointegration pathways, emphasizing the need for personalized treatment plans to improve patient outcomes.

## 1. Introduction

Dental implants have become a widely accepted solution for restoring function and aesthetics in patients with tooth loss. However, the success of dental implants largely depends on the patient’s systemic health, which is precisely the condition that affects bone metabolism and healing capacity. Osteoporosis and diabetes influence bone quality and healing, presenting potential challenges for implant stability and longevity [1,2].

The success of dental implants depends on their stability, which can be classified into two distinct stages: primary stability and secondary stability. Primary stability represents the initial mechanical stability of the implant, achieved immediately after insertion, and is influenced by bone density and quality, implant design, and the surgical technique used. This stage is essential for preventing implant movement during initial healing. In contrast, secondary stability reflects the biological process of osseointegration, by which bone forms and adheres to the implant surface, resulting in long-term stability. This becomes predominant as primary stability decreases during bone remodeling. Understanding and assessing both stages is essential for optimizing implant success, especially in patients with systemic conditions that affect bone quality, such as diabetes and osteoporosis.

Osteoporosis is characterized by decreased bone density and deterioration of bone microarchitecture, increasing the risk of fractures and bone loss [3]. Studies have shown that patients with osteoporosis may experience reduced osseointegration of dental implants, as compromised bone quality can impair the stability needed for successful implant integration [4]. Bisphosphonate treatment is commonly used to manage osteoporosis, yet it can also present risks, such as osteonecrosis of the jaw, which may complicate implant therapy in these patients [5].

Osteoporosis is characterized by reduced bone density and microstructural deterioration, increasing fragility and fracture risk. This issue poses a significant challenge in dental implants, as low bone quality might impact the implant’s main stability, which is essential for proper osseointegration [6].

The quality and amount of bone in which the implant is positioned define primary implant stability, and osteoporosis impairs both factors, decreasing the bone’s capacity to sustain the implant in the early phases of healing. Furthermore, osteoporosis patients frequently have poor bone remodeling processes, which can postpone osseointegration and raise the risk of marginal bone loss and implant movement [7,8,9].

On the other hand, diabetes mellitus, particularly when uncontrolled, poses a different set of challenges. High blood glucose levels contribute to chronic inflammation, impaired immune response, and delayed wound healing [6]. In patients with diabetes, the risk of peri-implantitis and implant failure is notably higher, as hyperglycemia can alter the bone healing process and reduce the bone quality surrounding the implant [7]. Additionally, the presence of inflammatory mediators in diabetic patients can compromise implant stability and increase the risk of implant mobility over time [8].

Osteoporosis reduces bone density and quality, compromising primary stability for implant integration [5]. Studies have shown that patients with osteoporosis are at increased risk of implant mobility and marginal bone loss, which can lead to implant failure in the early stages [6]. According to some research, using osseodensification techniques and resonance frequency analysis (RFA) can contribute to increasing the stability of implants in these patients [7,8,9].

Diabetes impairs bone healing and remodeling, predisposing patients to delayed healing and a higher risk of peri-implant infections [8]. This is particularly problematic for osseointegration, causing an increase in implant mobility. According to a study by Zhang et al., type 2 diabetes can lead to long-term implant instability, affecting peri-implant bone quality [9]. RFA and radiographic methods are frequently used to assess implant stability in these patients [10,11,12,13,14].

Diabetes is a chronic metabolic condition characterized by persistent hyperglycemia, which impairs the body’s ability to heal and regenerate tissues, including bone. In dental implants, diabetes, especially if poorly controlled, is a significant risk factor that can compromise implants’ long-term stability and success [15].

One of the main problems associated with diabetes is delayed wound healing. Hyperglycemia impairs the function of cells involved in bone regeneration, such as osteoblasts, and reduces local blood flow, which can delay bone formation and remodeling around the implant [16]. In addition, diabetes is associated with chronic low-grade inflammation, which can increase bone resorption and increase the risk of peri-implant infections, such as peri-implantitis [17].

Diabetes directly affects osseointegration, the process of biological bonding between bone and implant, which is critical for achieving secondary implant stability. Chronic inflammation, hyperglycemia, and delayed tissue healing associated with diabetes may compromise this process, increasing the risk of implant instability. This increases the risk of marginal bone loss, implant mobility, and treatment failure, especially in patients with poor glycemic control [18,19].

However, studies show that diabetic patients can benefit from dental implants if blood sugar is well managed before and after the intervention. Careful treatment planning, advanced implant stabilization techniques (such as osseodensification), and constant monitoring of blood sugar levels are essential for success [15,18]. Also, using modern tools, such as resonance frequency analysis (RFA), allows the doctor to follow the implant’s long-term stability and adapt the treatment according to the patient’s specific needs [20].

Thus, although diabetes is a challenge, it does not preclude the success of dental implant treatment, provided that a multidisciplinary approach is adopted and increased attention is paid to managing the patient’s overall health.

Frequency analysis, radiography, and advanced biomechanical parameters are essential to evaluating the stability of implants in patients with these conditions [11,12]. Integrating these methods into a systematic monitoring plan could allow early detection of stability problems and prompt treatment adjustment [13].

Understanding how osteoporosis and diabetes affect bone metabolism and healing is critical for optimizing dental implant stability and patient outcomes. Previous studies have investigated these conditions individually [11]; however, a comprehensive comparative study evaluating their relative impact on implant mobility and stability over time is essential to guide clinical decision-making [9,10].

Thus, this study aims to evaluate the influence of osteoporosis and diabetes on dental implant stability through a comparative analysis. It will focus on identifying specific risk factors associated with each condition that may contribute to increased implant mobility.

This study compares osteoporosis and diabetes, two systemic conditions with different etiologies that affect bone quality and healing. It provides valuable insights into the challenges and strategies for optimizing the success of dental implants. Even though osteoporosis and diabetes have distinct pathological mechanisms, both lead to alterations in bone quality that influence implant stability, which justifies a comparative analysis.

While the etiologies differ, this study focuses on understanding how each condition uniquely impacts implant stability. The findings will help clarify whether patients with osteoporosis or diabetes exhibit significantly different outcomes in implant stability, providing valuable insights for tailored treatment planning in these populations.

## 2. Materials and Methods

This research aims to evaluate the mobility of dental implants in patients with systemic conditions (diabetes and osteoporosis) at 6 and 12 months after their insertion, using objective measurement methods to compare their stability according to the underlying condition.

This study included 50 patients (n = 50) eligible for dental implant treatment, selected based on specific systemic conditions. The patients were divided into two groups: the group A diabetes group, 21 patients diagnosed with type 2 diabetes (HbA1c controlled below 7.5% for at least 6 months before the implant insertion procedure), and the group with osteoporosis group B, 29 patients diagnosed with osteoporosis (confirmed by DEXA bone densitometry, T-score ≤ −2.5).

The patients have consented to this study, which Apollonia University’s Ethics Committee approved under reference number 21 on 5 October 2023.

Inclusion criteria included adults aged 45–75 with stable general health, no severe systemic conditions, successful implant insertion, and no perioperative complication.

The exclusion criteria were patients with other major metabolic or autoimmune diseases and dental implant patients with significant postoperative complications or requiring implant revision during the study period. Postoperative complications observed during the study included peri-implant infections, significant marginal bone loss, and excessive implant mobility, which required revision. These complications were more common in patients with diabetes due to insufficient glycemic control and in those with osteoporosis due to low bone density.

This study is a prospective cohort type, in which the mobility of the implants is measured at two postoperative intervals, respectively, 6 months and 12 months after implant insertion. The stability comparison between the two groups aims to identify the influence of diabetes and osteoporosis on dental implant stability during the first 12 months. All patients benefited from implant insertion according to the standard protocol, according to bone density, without immediate implant loading. Implant design and material type were standardized to minimize variability. The implants used in the study were grade IV titanium with an anodically treated surface. They had a conical design, a length between 10 and 13 mm, and a variable diameter between 3.5 and 4.5 mm, adapted depending on bone density and anatomical location.

Implant mobility was assessed using Osstell ISQ (resonance frequency analysis for ISQ scores, Gothenburg, Sweden) and Periotest M (Medizintechnik Gulden, Modautal, Germany, return time to initial position for PTV values). Osstell ISQ measures secondary stability by frequency resonance analysis and provides ISQ values between 55–85. Periotest M evaluates implant mobility by measuring the time to return to the initial position, resulting in PTV values between −8 and 0. Osstell ISQ is non-invasive and allows long-term osseointegration monitoring but can be influenced by sensor positioning. Periotest M is simple to use and affordable but can be affected by bone density and implant loading. Both methods provide complementary information for implant research, contributing to a detailed assessment of implant stability [1].

Thus, secondary stability was assessed 6 months post-implantation using Osstell ISQ to analyze frequency resonance and obtain a numerical ISQ score. Periotest M was used to measure implant mobility, expressed in PTV values. Secondary stability was measured at 12 months, using the same methods, to monitor the progress of osseointegration. ISQ values between 55 and 85 were considered satisfactory for stability, and PTV values between −8 and 0 indicated optimal mobility, reflecting adequate osseointegration.

ISQ and PTV data were recorded and compared between the two groups at 6 months and 12 months to observe variations in implant stability between diabetic and osteoporotic patients.

### Statistical Analysis

To analyze the differences between the two groups at each time interval, we used appropriate statistical tests: Student’s *t*-test for independent samples, or the Mann–Whitney test for non-parametric data, depending on the distribution of the data. The evolution of implant stability between 6 and 12 months within each group was evaluated by paired tests, specifically the *t*-test for paired samples. A significance threshold of *p* < 0.05 was considered relevant to detect significant differences between groups and intervals. Statistical analyses were performed using IBM SPSS Statistics (version 26, IBM Corp., Armonk, NY, USA).

## 3. Results

### 3.1. Baseline Characteristics of Patients

Table 1 indicates the baseline characteristics of the studies. In the diabetes group, there was a balanced gender distribution, with ten males (47.6%) and 11 females (52.4%). The osteoporosis group had a higher proportion of females, with 12 males (41.4%) and 17 females (58.6%). Overall, females constituted 58% of the study population, possibly reflecting osteoporosis prevalence trends.

The average age in the diabetes group was 58 years (±6.2), while the osteoporosis group had a slightly higher mean age of 61 years (±5.8). This age range (approximately 45–75 years) represents a demographic at greater risk for bone metabolism disorders, relevant for implant stability considerations. A more significant proportion of patients in both groups resided in urban areas: 14 urban and 7 rural in the diabetes group, and 19 urban and 10 rural in the osteoporosis group. The overall urban representation was 66%, highlighting a potentially higher demand for implant treatments in urban settings. Table 1 shows the distribution of smokers and non-smokers by bone density categories within diabetes and osteoporosis groups. The prevalence of smoking, significant in both the diabetic (36%) and osteoporosis (34%) groups, highlights its negative impact on dental implant osseointegration by reducing local vascularization and impairing bone regeneration. However, the comparable percentages of smokers in both groups enable a fair comparison of the systemic effects of diabetes and osteoporosis on implant stability, minimizing smoking as a confounding factor. Implants were distributed between the maxilla and mandible. In the diabetes group, 12 implants were placed in the maxilla and 9 in the mandible. Similarly, 16 implants were in the maxilla and 13 in the mandible in the osteoporosis group.

This 56% preference for maxillary placement is common in dental implant studies, as variations in upper bone density may affect implant stability. Bone density varied across patients, with “low” bone density more common in the osteoporosis group (31%) than in the diabetes group (24%). Moderate and high bone densities were more evenly distributed, with 28% of patients exhibiting high bone density across both groups. This may be relevant in assessing implant stability differences based on underlying bone quality.

### 3.2. Measurements in Patients with Osteoporosis

The analysis of implant stability in patients with osteoporosis was conducted at two postoperative intervals: 6 and 12 months, Table 2. Measurements were taken using the Osstell ISQ (for resonance frequency, which reflects implant stability) and the Periotest M (which measures implant mobility). The mean ISQ score increased from 67.04 at 6 months to 68.93 at 12 months, indicating a general improvement in stability over time. The increase in ISQ score was not statistically significant (*p* = 0.1960), indicating that while there was an improvement, it may not be large enough to confirm a significant change in stability.

The mean PTV score improved from −2.25 at 6 months to −3.34 at 12 months, reflecting reduced mobility and enhanced stability during the follow-up period. This improvement in PTV score was statistically significant (*p* = 0.0068), suggesting a meaningful increase in implant stability from 6 to 12 months for osteoporotic patients.

While ISQ and PTV measurements show improved implant stability over time, the statistically significant PTV results indicate a measurable stability enhancement for osteoporotic patients. The ISQ results align with this trend but do not reach statistical significance, possibly due to the small sample size or individual variations in patient bone integration rates.

### 3.3. Measurements in Patients with Osteoporosis

The analysis evaluates implant stability in diabetic patients at 6 and 12 months post-implantation, using ISQ (Osstell) and PTV (Periotest) measurements shown in Table 3.

The average ISQ score increased from 62.10 at 6 months to 65.79 at 12 months, showing a positive trend in implant stability over time. This change was statistically significant (*p* = 0.0215), indicating that diabetic patients experienced a measurable improvement in implant stability from 6 to 12 months, suggesting successful osseointegration despite the systemic challenges of diabetes.

The average PTV score improved from −1.06 at 6 months to −2.81 at 12 months, indicating reduced implant mobility and enhanced stability over time. This change was highly statistically significant (*p* = 0.0001), underscoring a substantial gain in strength, with the implant reaching a more stable position as bone integration progressed.

ISQ and PTV measurements significantly improve implant stability between the 6- and 12-month marks in diabetic patients. This indicates successful implant integration, although monitoring is crucial given the potential effects of diabetes on bone healing and inflammation. These findings suggest that, with careful management, diabetic patients can achieve stable outcomes for dental implants.

The negative PTV values observed in this study indicate good implant stability in osteoporosis and diabetes groups. At 12 months, more negative values reflect improved osseointegration and reduced implant mobility.

### 3.4. Comparative Measurements in Patients with Osteoporosis and Patients with Diabetes

The mean ISQ score at 6 months was 67.04, suggesting a moderate level of initial implant stability (Table 4). The diabetes group had a lower mean ISQ score of 62.10, indicating lower initial stability than the osteoporosis group. The higher ISQ score in the osteoporosis group suggests better initial implant integration. However, both groups are within the range considered acceptable for stability.

The mean PTV score was −2.25, indicating low implant mobility and a positive stability outcome (Table 4). The mean PTV score was −1.06, indicating slightly higher mobility than the osteoporosis group. The osteoporosis group shows better initial stability based on lower mobility scores, which is favorable for implant success. The somewhat higher mobility in the diabetes group may reflect the effects of systemic health challenges on early-stage osseointegration.

The ISQ score at 12 months improved to 68.93, showing a modest increase in stability Table 5.

The diabetes group also showed improvement, with the ISQ score increasing to 65.79. Both groups demonstrate improved stability at 12 months, with the diabetes group showing statistically significant growth (*p* = 0.0215). This indicates successful progression in osseointegration for diabetic patients over time.

The PTV score improved to −3.34, indicating a further reduction in implant mobility and enhanced stability (Table 5). Similarly, the diabetes group saw an improved PTV score of −2.81. Both groups experienced statistically significant improvements in PTV scores, with the osteoporosis group reaching better stability overall. This suggests that, despite initial differences, both groups achieve notable stability by 12 months, indicating effective osseointegration.

## 4. Discussions

This study highlights the distinct effects of diabetes and osteoporosis on osseointegration by comparing dental implant stability in these patients. The results were analyzed using objective techniques like ISQ and PTV, which revealed that while both groups demonstrated notable improvements over 6 to 12 months, the underlying causes and extent of the changes varied. Implant stability measurements were not performed immediately after implantation to avoid mechanical or biological interference that could compromise the initial osseointegration process. Stability assessments were performed 6 and 12 months post-implantation, reflecting the natural progression of osseointegration and secondary stability of the implants.

Osteoporotic patients exhibited higher baseline ISQ values than diabetic patients, reflecting superior secondary stability. Low bone density, however, is known to increase the risk of decreased osseointegration [1,9,11]. According to research by Chrcanovic et al. and Zhang et al. [1,9,12,13], osteoporosis raises the risk of implant mobility, particularly if proper treatment is not received. Furthermore, as Ballantyne and De Medeiros et al. [13,14] noted, pharmaceutical therapies like bisphosphonates may hinder bone integration by increasing the risk of osteonecrosis.

Additionally, individuals with osteoporosis have shown increased implant stability when using sophisticated implant procedures such as osseodensification and resonant frequency monitoring [7,8,15,16,17,18,21]. These strategies have also been verified in animal settings, where beneficial clinical outcomes have been found when procedures are adapted based on bone properties [15]. Lower initial ISQ values in diabetic individuals indicate the detrimental effects of long-term inflammation and high blood sugar on bone repair [3,4,19,20,22].

This study’s results are consistent with Monje et al.’s findings, who reported an association between diabetes, peri-implantitis risk, and delayed healing [22,23]. However, as demonstrated by Naujokat et al. and Wagner et al. [3,17], a notable improvement in ISQ and PTV levels at 12 months suggests that proper glycemic control can lessen these adverse consequences. Furthermore, recent research has demonstrated that implants with anodized surfaces, which promote cell adhesion and lower the risk of inflammation, can positively impact osseointegration in diabetes patients [24,25,26,27]. These findings support the necessity of tailoring treatment methods to the patient’s metabolic circumstances. In contrast, diabetic patients improved more quickly up to 12 months (−2.81 PTV vs. −3.34), while osteoporotic patients demonstrated more excellent baseline stability (67.04 vs. 62.10 ISQ at 6 months) [19,24,27]. The benefits of glycemic management in the diabetic group and the better baseline bone quality in the osteoporosis group can be blamed for these discrepancies [28,29,30,31].

Research like that conducted by Oates et al. and Tsolaki et al. has demonstrated how important customized therapies are for implant stability [7,11]. This implies that pharmacological management, implant surface selection, and other aspects of therapy tailoring may lessen between-group differences and enhance clinical results. Aguilar-Salvatierra et al. and Simpson et al., for instance, showed that instantly loaded implants, when utilized carefully in aesthetic areas, can yield positive effects even when there are systemic concerns [23,24].

The results of our study highlight the determining role of systemic conditions on the osseointegration process of dental implants. However, it is important to explore the interaction between pharmacological treatments for osteoporosis or diabetes and the biological mechanisms involved in bone healing. For example, the long-term effect of bisphosphonates or antiresorptive therapies on osseointegration requires further studies [13].

In addition to osteoporosis and diabetes, other factors, such as nutritional status, vitamin D levels, or chronic inflammation, could contribute to variability in implant stability. Understanding these influences could improve clinical approaches and personalize treatment for each patient [15,19].

Recent studies highlight the importance of vitamin D supplementation in the success of dental implants, especially in patients with osteoporosis. Vitamin D is essential in the osseointegration process, facilitating the close and durable connection between implants and the bone tissue in which they are inserted [25].

The importance of an integrated management approach in patients with systemic conditions is emphasized. Using ISQ and PTV methods combined with advanced implantation techniques creates a reasonable basis for monitoring and optimizing treatments [27,28].

Using technologies such as osseodensification and implants with anodized surfaces opens new perspectives for optimizing dental implant outcomes in patients with systemic diseases. For example, anodized implants, which improve cell adhesion and reduce inflammation, have been associated with favorable outcomes in diabetic patients, according to recent studies [24,28]. In patients with osteoporosis, osseodensification has significantly improved secondary stability in clinical and animal studies [15,21,29].

Individuals with osteoporosis have also shown increased implant stability when sophisticated implant procedures, such as osseodensification and resonant frequency monitoring, are used [7,8,15,16,17,18,21].

While objective methods like Osstell ISQ and Periotest were used to assess implant stability, the study’s small sample size and 12-month follow-up period are notable limitations. Extending the follow-up period and including a larger sample could provide a more detailed understanding of the long-term differences between the two patient groups [9,10,30].

Future research should focus on randomized trials to evaluate the effectiveness of tailored treatment strategies for patients with diabetes and osteoporosis. In addition, using salivary biomarkers to monitor the osseointegration process, as suggested in other recent studies, could represent a non-invasive and effective method for assessing the risks associated with these conditions [22,23,31,32,33].

Bone augmentation techniques, such as bone addition and sinus lift, are essential for improving the stability of dental implants in patients with diabetes or osteoporosis. These procedures restore bone volume and density, ensuring adequate support for implants and contributing to their long-term success, even in conditions of compromised healing [34,35].

This comparison between two different systemic diseases highlights their unique effects on osseointegration. Our results emphasize the need for personalized therapeutic strategies, as osteoporosis and diabetes have distinct effects on osseointegration. They highlight their importance in ensuring the long-term success of dental implants while contributing valuable insights to the existing literature.

A limitation of the study is the absence of data on early (2–4 weeks) and intermediate (3–6 months) stability. These critical intervals were not included to avoid repeated mechanical interference, which could have compromised the osseointegration process. However, measurements taken at 6 and 12 months reflect the progression of late stability and are relevant to the clinical success of implants. Future research should include all stability stages (early, intermediate, and late) to provide a complete understanding of implant performance.

## 5. Conclusions

This study underscores the substantial impact of osteoporosis and diabetes on dental implant stability, highlighting the importance of personalized treatment strategies to enhance long-term outcomes. Future studies should focus on larger cohorts and longer follow-up periods to further explore the distinct effects of systemic conditions on implant success and to refine personalized treatment protocols.

## Figures and Tables

**Table 1:** Baseline characteristics.

Characteristic	Diabetes Group		Osteoporosis Group		Total (%)	
Gender	Male	Female	Male	Female	Male	Female
	10	11	12	17	42%	58%
Age	58 ± 6.2 years		61 ± 5.8 years			
Residence	Urban	Rural	Urban	Rural	Urban	Rural
	14	7	19	10	66%	34%
Smoking Status	Smoker	Non-Smoker	Smoker	Non-Smoker	Smoker	Non-Smoker
	8	13	10	19	36%	64%
Implant Location	Maxillary	Mandible	Maxillary	Mandible	Maxillary	Mandible
	12	9	16	13	56%	44%
**Bone Density**	**Smokers (Diabetes Group)**		**Non-Smokers (Diabetes Group)**	**Smokers (Osteoporosis Group)**		**Non-Smokers (Osteoporosis Group)**
Low	2		3	3		6
Moderate	4		6	4		8
High	2		4	3		5

**Table 2:** Comparison of implant stability measurements in osteoporotic patients at 6 and 12 months.

Measurement	6 Months (Mean ± SD)	12 Months (Mean ± SD)	t	*p*-Value
ISQ Score (Osstell)	67.04 ± 5.42	68.93 ± 4.83	−1.32	0.1960
PTV Score (Periotest)	−2.25 ± 1.39	−3.34 ± 1.59	2.92	0.0068

**Table 3:** Comparison of implant stability measurements in diabetic patients at 6 and 12 months.

Measurement	6 Months (Mean ± SD)	12 Months (Mean ± SD)	t	*p*-Value
ISQ Score (Osstell)	62.10 ± 5.46	65.79 ± 3.87	−2.49	0.0215
PTV Score (Periotest)	−1.06 ± 1.21	−2.81 ± 1.44	4.75	0.0001

**Table 4:** Osteoporosis and diabetes measurements—6 Months.

Measurement	Osteoporosis 6 Months (Mean ± SD)	Diabetes 6 Months (Mean ± SD)	*p*-Value
ISQ Score (Osstell)	67.04 ± 5.42	62.10 ± 5.46	0.1960
PTV Score (Periotest)	−2.25 ± 1.39	−1.06 ± 1.21	0.0001

**Table 5:** Osteoporosis and diabetes measurements—12 Months.

Measurement	Osteoporosis 12 Months (Mean ± SD)	Diabetes 12 Months (Mean ± SD)	*p*-Value
ISQ Score (Osstell)	68.93 ± 4.83	65.79 ± 3.87	0.1960
PTV Score (Periotest)	−3.34 ± 1.59	−2.81 ± 1.44	0.0001

## Data Availability

The original contributions presented in this study are included in the article. Further inquiries can be directed to the corresponding authors.
